# Liver Is Able to Activate Naïve CD8^+^ T Cells with Dysfunctional Anti-Viral Activity in the Murine System

**DOI:** 10.1371/journal.pone.0007619

**Published:** 2009-10-30

**Authors:** John R. Lukens, Joseph S. Dolina, Taeg S. Kim, Robert S. Tacke, Young S. Hahn

**Affiliations:** Beirne Carter Center for Immunology Research, Department of Microbiology, University of Virginia, Charlottesville, Virginia, United States of America; New York University School of Medicine, United States of America

## Abstract

The liver possesses distinct tolerogenic properties because of continuous exposure to bacterial constituents and nonpathogenic food antigen. The central immune mediators required for the generation of effective immune responses in the liver environment have not been fully elucidated. In this report, we demonstrate that the liver can indeed support effector CD8^+^ T cells during adenovirus infection when the T cells are primed in secondary lymphoid tissues. In contrast, when viral antigen is delivered predominantly to the liver via intravenous (IV) adenovirus infection, intrahepatic CD8^+^ T cells are significantly impaired in their ability to produce inflammatory cytokines and lyse target cells. Additionally, intrahepatic CD8^+^ T cells generated during IV adenovirus infection express elevated levels of PD-1. Notably, lower doses of adenovirus infection do not rescue the impaired effector function of intrahepatic CD8^+^ T cell responses. Instead, intrahepatic antigen recognition limits the generation of potent anti-viral responses at both priming and effector stages of the CD8^+^ T cell response and accounts for the dysfunctional CD8^+^ T cell response observed during IV adenovirus infection. These results also implicate that manipulation of antigen delivery will facilitate the design of improved vaccination strategies to persistent viral infection.

## Introduction

The liver is a site for continual exposure to bacterial constituents and food antigens. To prevent the generation of harmful immune responses against bacteria or innocuous food antigens, the liver evolves a way to dampen host immunity [1]. The tolerogenic nature of the liver is manifested by the high success rate of liver transplantation such that Iiver transplantation across MHC class I mismatches allows for successful acceptance of allografts [2], [3], [4]. In addition, the liver has been reported to be involved in the accumulation and deletion of activated CD8^+^ T cells: the removal of activated CD8^+^ T cells in the liver contributes to T cell homeostasis and the contraction of T cell responses during infection [5], [6], [7]. As a result of these properties, numerous hepatotropic infections including HCV, HBV, and malaria establish chronic infections in the liver compartment [8], [9], [10]. Notably, CD8^+^ T cell responses generated during viral infection in the liver with the establishment of viral persistence display a significant defect in effector function and become functionally exhausted in later stages of infection [11], [12]. Enhanced levels of T cell apoptosis have also been observed during chronic liver infection [13], [14].

Paradoxically, the liver is not solely the site to induce T cell tolerance. The liver appears to support robust effector CD8^+^ T cells responses against multiple pathogens [15], [16], [17]. For example, it has recently been shown that the liver is a major repository for effector CD8^+^ T cells during influenza infection [18], [19] and T cell mediated hepatocyte damage results from the liver infiltrating activated T cells [20], [21], [22]. It has been previously reported that the liver may serve as an extralymphoid site for T cell priming by utilizing model systems in which CD8^+^ T cell activation and priming were assessed following recognition of allogenic MHC molecules [23], [24], [25]. The strong intensity of TCR stimulation and high expression levels of MHC molecules during allogenic T cell activation do not accurately reflect what occurs during physiological anti-viral responses. Furthermore, CD8^+^ T cell activation can occur in the liver following recognition of non-self antigen [26]. However, it still remains unclear if primary T cell priming can occur in the liver following hepatotropic viral infection and if liver cells are able to present viral antigen and prime intrahepatic CD8^+^ T cells *in vivo*. Studies conducted to elucidate the immune factors involved in the effective generation and regulation of anti-viral CD8^+^ T cells to liver infections will greatly improve treatment strategies against persistent viral infections.

In this study, we sought out to determine the immunological factor(s) that play a pivotal role in altering the balance between T cell tolerance induction and immunity in the liver. We demonstrate here that the site of initial CD8^+^ T cell priming determines the generation of effector CD8^+^ T cell responses in the liver. Numerous studies have shown that IV adenovirus infection results in the delivery of recombinant viruses directly to the liver [27], [28] [29]. Here, we report that CD8^+^ T cell responses generated during IV adenovirus infection display a functional defect in effector cytokine production and CTL activity as observed during persistent liver infections. Interestingly, we demonstrate here that the liver can support CD8^+^ T cell priming; however, intrahepatic CD8^+^ T cell activation is suboptimal and does not result in the differentiation of effector CD8^+^ T cells. In contrast, when adenovirus infection of the liver is limited by delivering adenovirus via subcutaneous (SubQ) inoculation, potent anti-viral CD8^+^ T cells are detected in the liver. During SubQ adenovirus infection, CD8^+^ T cells are activated in the inguinal lymph node and differentiate into effector CD8^+^ T cells. However, effector CD8^+^ T cells that are primed in the periphery are rapidly suppressed following intrahepatic antigen recognition during viral infection. These results suggest that intrahepatic antigen presentation limits anti-viral CD8^+^ T cell responses both at the priming and effector stages of the T cell response.

## Materials and Methods

### Mice and Recombinant Adenovirus

Thy1.2^+^ C57BL/6 mice were purchased from Taconic Farms (Hudson, NY). Both Thy1.1^+^ and OT-1 mice were purchased from The Jackson Laboratory (Bar Harbor, ME). Splenectomized and sham C57BL/6 mice were purchased from Taconic Farms (Hudson, NY). OT-1 mice were bred with Thy1.1^+^ mice to generate the Thy1.1^+^OT-1^+^ mice. All mice were housed in a pathogen-free facility and were tested routinely for mouse hepatitis virus and other pathogens. All mice were handled according to protocols approved by the University of Virginia Institutional Animal Care and Use Committee.

Replication-deficient recombinant adenovirus (rAd) expressing the βgal protein under the control of the human CMV promoter (Ad-LacZ) and lacking E1 and E3 genes was provided by Dr. Greg Helm (University of Virginia). rAd expressing ovalbumin (Ad-OVA) was generously provided by Dr. Timothy L. Ratliff (University of Iowa) and the Iowa Gene Transfer Vector Core (Iowa City, IA). Adenovirus vectors were propagated in 293A cells and purified by two consecutive cesium chloride gradient. Mice were injected with 5×10^8^, 5×10^7^, 5×10^6^, or 5×10^5^ PFU of rAd per mouse by either SubQ or IV immunization route depending on the experiment.

### Liver Leukocytes Isolation and Flow Cytometric Analysis

Intrahepatic lymphocytes (IHLs) were isolated from livers as described previously [30]. Briefly, the liver was perfused with PBS/0.05% collagenase (Sigma-Aldrich) and then washed with Iscove's Modified Dulbecco's Medium (IMDM) supplemented with 10% newborn calf serum (NBCS). Liver sections were finely minced and digested further with PBS/0.05% collagenase. Mononuclear cells were purified by nycodenz gradient centrifugation. Splenocytes were prepared by mechanical disruption and isolation over a Ficoll gradient.

PE-labeled H2-K^b^ βgal tetramer (ICPMYARV) was provided by the Tetramer Core Facility of the National Institutes of Health. APC-labeled H2-K^b^ OVA tetramer (SIINFEKL) was purchased from the Baylor College of Medicine MHC Tetramer Core Laboratory. The following reagents were used for cell surface and intracellular staining: anti-CD8 (clone 53-6.7), anti-PD-1 (clone J43), anti-B7-H1 (clone MIH5), anti-IFN-γ (clone XMG1.2), anti-TNFα (clone MP6-XT22), anti-CD25 (clone PC61), anti-CD69 (clone H1.2F3), anti-CD62L (clone MEL-14), anti-Thy1.1 (clone HIS51), anti-Thy1.2 (clone 53-2.1), anti-MHC-II (clone M5/114.15.2), and anti-CD11c (clone N418) Abs which were purchased from eBioscience (San Diego, CA). Anti-granzyme B (clone GB12) Ab was purchased from Caltag Laboratories (Burlingame, CA). Anti-CD8 (clone 53-6.7) and anti-Thy1.1 (clone OX-7) Abs were purchased from BD Bioscience (San Jose, CA). For the cell surface labeling experiments, 2×10^6^ splenocytes or liver leukocytes were incubated with the corresponding Abs and tetramer for 30 min at 4°C in staining buffer (PBS with 2% FBS and 0.1% NaN_3_). After staining, cells were fixed in BD FACS lysing solution. Flow cytometry data for each of the experiments was acquired using a BD FACS Canto (BD Immunocytometry Systems). Results were analyzed using FlowJo software (Tree Star Inc., Ashland, OR).

### Adoptive Transfer of TCR Transgenic T Cells

CD8^+^ T cells were isolated from the spleens of Thy1.1^+^OT-1^+^ mice using positive magnetic bead separation (Miltenyi Biotec). Greater than 95% of the splenic CD8^+^ T cells that were isolated from the Thy1.1^+^OT-1^+^ mice were specific for the OVA_257–264_ epitope and stained positive for OVA tetramer before adoptive transfer (data not shown). In the experiments that were conducted to assess T cell proliferation, purified CD8^+^ T cells were labeled with 1.8 µM CFSE, and CFSE labeled Thy1.1^+^OT-1^+^CD8^+^ T cells (2×10^6^ cells per mouse) were adoptively transferred by tail vein injection into naïve Thy1.2^+^ mice. Mice were allowed to rest for one day and then mice were infected by either IV or SubQ immunization route with Ad-OVA. In the experiments that utilized splenectomized mice to ascertain liver specific priming of CD8^+^ T cells, mice were treated with Mel-14 Ab (100 µg/mouse) by IP administration one day before adoptive transfer to limit the entry of Thy1.1^+^OT-1^+^CD8^+^ T cells into the LNs. To verify efficient blockade of T cell entry into LNs following MEL-14 Ab treatment, the presence of adoptively transferred Thy1.1^+^OT-1^+^CD8^+^ T cells in the inguinal lymph nodes (Ig LN) was evaluated. For the early activation studies, purified 4×10^6^ Thy1.1^+^OT-1^+^CD8^+^ T cells were adoptively transferred by tail vein injection into naïve Thy1.2^+^ mice. Mice were allowed to rest for one day and then were infected with Ad-OVA. Organs were harvested at 4, 6, and 24 hours post-infection (p.i.) and the expression of CD69, CD25, and OVA tetramer by antigen-specific and endogenous CD8^+^ T cells was analyzed by flow cytometry. For the 0 hour data, mice were treated with PBS as a control.

### Ex Vivo Analysis of Ag-Specific CD8^+^ T Cell Function

2×10^6^ splenocytes or liver leukocytes were incubated for 5 hours in IMDM supplemented with 10% FBS, 10 U/ml penicillin G, 2 mM L-glutamine, 0.05% 2-ME, 1 µg/mL brefeldin-A (BD Biosciences) and 2 µg/ml of either OVA (SIINFEKL) peptide or LacZ (ICPMYARV) peptide. For the PMA/ionmycin re-stimulation assay, 500 ng/mL ionomycin, 5 ng/mL PMA, and 1 µg/mL brefeldin-A (BD Biosciences) was added to re-stimulation media instead of peptide. After incubation, the cells were surface labeled with anti-CD8 Ab as described earlier, followed by intracellular staining for IFN-γ, granzyme-B, and TNF-α. For the antigen presentation assay, Thy1.2^+^ C57BL/6 mice were infected with 5×10^8^ PFU Ad-OVA via either SubQ or IV immunization. At 48 hours p.i., liver, spleen, Ig LN, and liver LN cells were isolated and then incubated with 1×10^5^ naïve CFSE labeled Thy1.1^+^OT-1^+^CD8^+^ T cells in vitro for 72 hours.

### Effect of Liver Antigen on Influencing Intrahepatic CD8^+^ T Cell Effector Function

5×10^6^ Thy1.1^+^OT-1^+^CD8^+^ T cells were adoptively transferred into Thy1.2^+^ C57BL/6 mice followed by infection with 5×10^8^ PFU Ad-OVA via SubQ inoculation one day later. On day 4 p.i., spleen and Ig LN cells were isolated from these mice and Thy1.1^+^ cells were purified using positive magnetic bead separation. 2×10^6^ Thy1.1^+^OT-1^+^CD8^+^ T cells from day 4 SubQ primed mice were adoptively transferred into Thy1.2^+^ mice that were infected for 4 days earlier via IV inoculation with either 5×10^8^ PFU Ad-LacZ (inflammation group) or 5×10^8^ PFU Ad-OVA (Ag+inflammation group). As a control, 5×10^6^ Thy1.1^+^OT-1^+^CD8^+^ T cells from day 4 SubQ primed mice were adoptively transferred into naïve Thy1.2^+^ mice (control group). At 24 hours post adoptive transfer, CD8^+^ T cell responses were analyzed by flow cytometry.

### Generation of Bone Marrow Chimeras

C57BL/6 recipient mice were lethally irradiated (950 rad) and were then injected IV with 5×10^6^ bone marrow cells from C57BL/6 or BALB/c mice that were depleted of T cells using magnetic bead separation (Miltenyi Biotec) according to the manufacturer's protocol. To limit pathogenic infection during bone marrow reconstitution, mice were given 1 mg/ml sulfadoxin in the drinking water during the first month. Chimeric mice were used in experiments at least 2 months after reconstitution. The expression of H2-K^b^ and H2-K^d^ by CD11b^+^CD45^+^ cells in the liver was determined using flow cytometry to verify complete donor chimerism.

### Statistical Analysis

Student's *t* tests were used to evaluate the significance of the differences. A value of *p*<0.05 was regarded as statistically significant.

## Results

### Reduced CD8^+^ T Cell Responses Are Generated by Intravenous Administration of Adenovirus

Secondary lymphoid tissues, e.g. lymph nodes and spleen, are generally considered to be the major sites of activation, proliferation, and differentiation of naïve antigen-specific CD8^+^ T cells into effector cells. However, the liver has also been implicated as a potential site of extralymphoid induction of CD8^+^ T cell responses to alloantigens and hepatotropic infectious agents including viruses. Recombinant adenoviruses (rAd) have been demonstrated to direct expression of foreign antigens to the liver when rAd is administered by the intravenous (IV) route. To examine whether intrahepatic expression of foreign antigen delivered by rAd vector would result in the activation of antigen specific CD8^+^ T cells and the subsequent development of CD8^+^ effector T cells, we infected C57BL/6 mice with 5×10^8^ PFU of rAd expressing the gene encoding ovalbumin (OVA) protein (Ad-OVA) by IV route. We chose Ad-OVA to probe the CD8^+^ T cell response to the liver because this protein induces a robust CD8^+^ T cell response in this mouse strain and OVA antigen-specific T cells from TCR transgenic mouse lines are available for more detailed analysis on antigen-specific CD8^+^ T cell responses. As a control, we introduced the same inoculum dose of Ad-OVA by subcutaneous (SubQ) route in order to initiate CD8^+^ T cell response through secondary lymphoid tissue presentation within the lymph nodes draining the site of virus infection.

**Figure 1:**
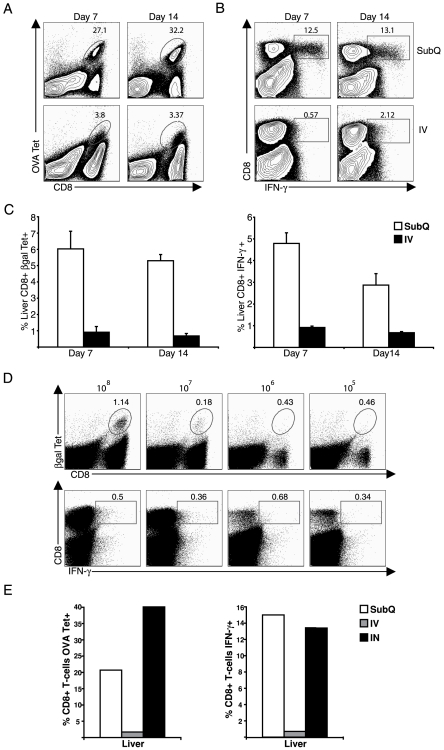
IV adenovirus administration results in the diminished CD8^+^ T cell responses in the liver. (A, B) C57BL/6 mice were infected with 5×10^8^ PFU Ad-OVA via either SubQ or IV inoculation. At days 7 and 14 p.i., liver leukocytes were isolated and the percentage of OVA tet^+^CD8^+^ T cells (A) was determined by direct *ex vivo* staining. The percentage of CD8^+^ T cells producing IFN-γ (B) was assessed following OVA peptide restimulation for 5 hours in the presence of monensin. Data are representative of at least two independent experiments for each time point (*n* = 3/group). (C) C57BL/6 mice were infected with 5×10^8^ PFU Ad-LacZ via either SubQ or IV administration. At days 7 and 14 p.i., the percentage of βgal tet^+^CD8^+^ T cells and IFN-γ-producing CD8^+^ T cells was determined. Data are presented as averages ± SEM (*n* = 3/group). (D) C57BL/6 mice were infected with Ad-LacZ via IV inoculation at 10^8^, 10^7^, 10^6^, or 10^5^ PFU per mouse. The percentage of βgal tet^+^CD8^+^ T cells and IFN-γ^+^CD8^+^ T cells in the liver was determined at day 7 p.i. Data are representative of three independent experiments. (E) C57BL/6 mice were infected with 5×10^8^ PFU Ad-OVA via SubQ, IV, or IN immunization. At 7 days p.i., the percentage of OVA tet^+^CD8^+^ T cells and IFN-γ^+^CD8^+^ T cells resident in the liver was calculated. Data are representative of two independent experiments.

SubQ inoculation of virus resulted in the accumulation of a significant number of OVA-specific tet^+^ CD8^+^ T cells in the liver of inoculated mice at days 7 and 14 p.i. (Fig. 1A). Approximately 50% of OVA-specific tet^+^ CD8^+^ T cells in the liver produce IFN-γ in response to specific peptide stimulation *in vitro* (Fig. 1B). As demonstrated below, the accumulation of these CD8^+^ effector T cells in the liver of mice with SubQ infection reflects the activation/proliferation and differentiation of naïve antigen specific CD8^+^ T cells in the lymph nodes draining the site of virus infection with subsequent migration of the activated cells into the liver at later times. In contrast, the IV administration of virus resulted in a markedly diminished OVA specific CD8^+^ T cell response in the liver as detected by tetramer staining (Fig. 1A) with a likewise markedly diminished frequency of CD8^+^ T cells responding to specific peptide stimulation *in vitro* with IFN-γ production (Fig. 1B). This diminished response to IV adenovirus delivery was not an exclusive property of the Ad-OVA. When we carried out a corresponding comparison of the impact of IV versus SubQ inoculation on the subsequent CD8^+^ T cell response in the liver using rAd expressing β-galactosidase (Ad-LacZ), we observed a similar diminished CD8^+^ T cell response to IV administration of Ad-LacZ at days 7 and 14 p.i. as measured by both tetramer staining and IFN-γ production (Fig. 1C).

The finding of the reduced CD8^+^ T cell response to IV virus administration was not expected and suggested that there may be a diminished induction of antigen specific anti-viral CD8^+^ T cell responses and/or a defective response of effector T cells generated following IV virus administration when antigen expression is targeted to the liver. It should be noted, however, that IV administration of rAd has been reported to result in immunologic tolerance although the observation is made by administration of rAd virus at 10-100 fold higher doses than that employed in our studies [31]. To determine if the diminished CD8^+^ T cell response observed reflected a high tolerogenic dose of virus delivered by the IV route, we analyzed the effect for various doses of rAd virus on the generation of CD8^+^ T cell responses in the liver following IV virus inoculation. As Fig. 1D demonstrates, the decreased dose of virus inoculum administered by the IV route only served to reduce the overall magnitude of CD8^+^ T cell response detected in the liver by either tetramer staining or IFN-γ production.

We believe, therefore, that under the experimental conditions employed here, the diminished CD8^+^ T cell response observed in the liver after IV virus administration was not easily attributable to high dose tolerance based on the size of the inoculum employed. Rather the results (along with findings presented below) suggest that IV administration of this hepatotropic virus results in direct priming of CD8^+^ T cells in the liver with consequent alterations in CD8^+^ T cell activation and/or function. To further test the impact of inoculum dose and route of virus administration on the subsequent CD8^+^ T cell response, we inoculated mice with 5×10^8^ PFU of rAd by the intranasal (IN) route and evaluated the development of the CD8^+^ T cell response in the lungs and liver thereafter. As reported by others for virus infection in the respiratory tract [32], virus administration at this site results in induction of CD8^+^ T cell responses exclusively in the lymph nodes draining the respiratory tract. The IN administration of virus which, like SubQ virus administration, restricts virus to the lymph nodes draining the site of inoculation, results in a vigorous CD8^+^ T cell response in the liver (Fig. 1E) and lungs (data not shown).

### Induction of CD8^+^ T Cell Responses Can Occur in the Liver

The IV administration of virus will not only deposit virus in the liver but antigen can be detectable in other sites including the secondary lymphoid tissues by migration of dendritic cells taken up antigen. It was therefore important to demonstrate whether virus deposition in the liver resulted in CD8^+^ T cell activation in that site and whether altered CD8^+^ T cell responses in the liver could account for the observed impaired responses observed. One potential explanation for the finding of diminished CD8^+^ T cell responses in the liver following IV virus inoculation is that there might be a defect in the activation and/or proliferation of naïve CD8^+^ T cells responding directly in the liver. In order to determine if the liver could serve as a site of naïve CD8^+^ T cell activation following IV virus administration in addition to secondary lymphoid organs, we employed an adoptive transfer strategy using OT-1 CD8^+^ TCR transgenic T cells directed to the OVA protein epitope displayed by the Ad-OVA virus. These Thy1.1^+^ naïve CD8^+^ TCR transgenic T cells were labeled with the dilution sensitive dye CFSE and then adoptively transferred into congenic Thy1.2^+^ recipients. Upon 24 hours following transfer, the recipient mice received Ad-OVA virus by SubQ or IV route. Activation and proliferation of the OT-1 T cells in the liver, spleen, and lymph nodes was monitored by CSFE dilution and T cell activation marker expression at 36 and 48 hrs post virus inoculation.

**Figure 2:**
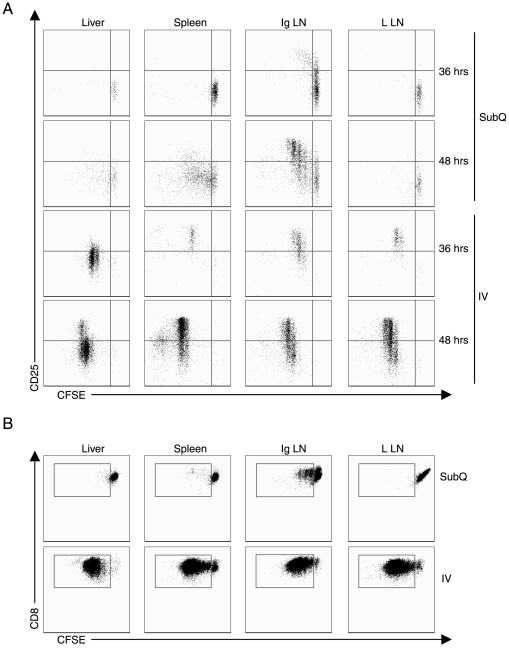
IV adenovirus administration leads to systemic CD8^+^ T cell proliferation. (A) CFSE labeled Thy1.1^+^OT-1^+^CD8^+^ T cells (2×10^6^ cells per mouse) were adoptively transferred into naïve Thy1.2^+^ C57BL/6 mice. After 24 hours of adoptive transfer, the recipient mice were then infected with 5×10^8^ PFU Ad-OVA via either SubQ or IV injection. At 36 and 48 hours p.i., the liver, spleen, Ig LN, and liver LN were isolated and the expression of CD25 by proliferating Thy1.1^+^CD8^+^ T cells was analyzed by flow cytometry. The plots are gated on Thy1.1^+^CD8^+^ T cells. Data are representative of three independent experiments. (B) Thy1.2^+^ C57BL/6 mice were infected with 5×10^8^ PFU Ad-OVA via either SubQ or IV inoculation. At 48 hours p.i., liver, spleen, Ig LN, and liver LN cells were isolated and then incubated with 1×10^5^ naïve CFSE labeled Thy1.1^+^OT-1^+^CD8^+^ T cells *in vitro* for 72 hours. The plots are gated on Thy1.1^+^CD8^+^ T cells and the presence of dividing Thy1.1^+^CD8^+^ T cells was determined. Data are representative of two independent experiments.

As Fig. 2A demonstrates, following SubQ virus administration, proliferation of the adoptively transferred OT-1 T cells was restricted primarily to the inguinal lymph nodes draining at the site of SubQ virus administration. As expected the activated proliferating T cells upregulated expression of CD25. The onset of T cell proliferation occurred between 36 and 48 hrs after virus inoculation. This time frame for proliferation presumably reflects the time required for uptake of virus by tissue antigen presenting cells (APC) and subsequent migration of the APC to the draining inguinal nodes for antigen presentation to naïve T cells. In addition, there was minimal proliferation of T cells present in the liver, spleen or non-draining lymph nodes over this 48 hrs time frame. Thus following SubQ virus administration, viral antigen presentation was restricted to the draining lymph nodes at the site of virus inoculation. We confirmed this conclusion in companion experiments where mononuclear cells were isolated from the liver, spleen, and draining and non-draining lymph nodes at 48 hours following SubQ Ad-OVA administration. These leukocytes were then co-cultured *in vitro* for 3 days with CSFE-labeled naïve OT-1 T cells. When cultured T cells were evaluated for activation/proliferation, only mononuclear cells isolated from the draining inguinal lymph nodes were able to trigger T cell proliferation *in vitro* (Fig. 2B).

When virus was delivered by IV route, by contrast, proliferating T cells were detected in the liver, spleen and lymph node compartments at 36 hours p.i. and proliferation proceeded over the liver and the secondary lymphoid tissues with similar kinetics over the succeeding 12 hours (Fig. 2A). This finding suggested that after IV inoculation, virus was distributed to both the liver and secondary lymphoid tissues. In keeping with this suggestion, we found that mononuclear cells isolated from both liver and secondary lymphoid organs could support the *in vitro* proliferation of naïve OT-1 T cells (Fig. 2B). The *in vivo* analysis of the T cell response to IV virus inoculation also revealed that while the activation marker CD25 was significantly upregulated in T cell responses within the secondary lymphoid organs, CD25 expression was blunted/suppressed in OT-1 T cells responding in the liver (Fig. 2B). Minor CD25^+^CD8^+^ T cell population appears at 48 hour possibly due to the migration of CD8^+^ T cells generated in the secondary lymphoid organs into the liver.

**Figure 3:**
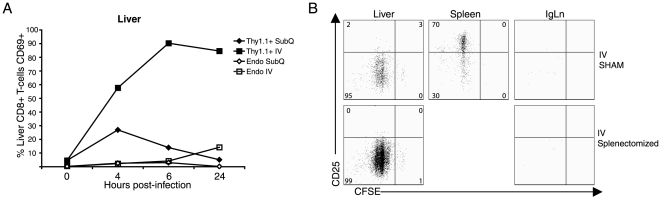
The liver supports CD8^+^ T cell priming with rapid activation of antigen-specific CD8^+^ T cells. (A) To determine how early CD8^+^ T cells are activated in the liver, 4×10^6^ Thy1.1^+^OT-1^+^CD8^+^ T cells were adoptively transferred into naïve Thy1.2^+^ C57BL/6 mice and then mice were infected with 5×10^8^ PFU Ad-OVA via either SubQ or IV administration one day later. At 4, 6, and 24 hours p.i., liver and Ig LN cells were isolated and the expression of CD69 by antigen specific CD8^+^ T cells (Thy1.1^+^OT-1^+^CD8^+^ gated) and endogenous CD8^+^ T cells (Thy1.1^−^CD8^+^ T cells) was analyzed by flow cytometry. The 0 hour data points indicate control mice that received PBS administration. Data are representative of at least two independent experiments. (B) 2×10^6^ CFSE labeled Thy1.1^+^OT-1^+^CD8^+^ T cells were adoptively transferred into either splenectomized or sham control Thy1.2^+^ C57BL/6 mice treated with Mel-14 Ab one day prior to adoptive transfer. The recipient mice were then infected with 5×10^8^ PFU Ad-OVA via IV administration one day later after adoptive transfer. At 48 hours p.i., liver, spleen, and IgLN cells were isolated and the expression of CD25 by proliferating Thy1.1^+^CD8^+^ T cells was analyzed by flow cytometry. The plots are gated on Thy1.1^+^CD8^+^ T cells and the numbers represent the percentage of cells within the indicated gates.

The above kinetic analysis suggested that following IV virus administration, the liver could support the initial activation and early proliferation of naïve antigen specific CD8^+^ T cells. However, we could not exclude the possibility that following IV virus inoculation, CD8^+^ T cells were activated in the secondary lymphoid organs, i.e. spleen and lymph nodes, and subsequently migrated to the liver. To further explore these alternatives, we first examined transferred OT-1 T cells resident in the liver for the expression of the early activation marker CD69 following IV or SubQ administration of Ad-OVA virus. As Fig. 3A demonstrates, upregulation of CD69 could be detected in the liver resident TCR transgenic CD8^+^ T cells as early as 4 hours following IV virus administration. As expected, minimal upregulation of CD69 was observed in liver resident OT-1 T cells up to 24 hours following SubQ virus inoculation. Importantly, non-transgenic antigen-nonspecific Thy1.2^+^ recipient endogenous CD8^+^ T cells present in the liver demonstrated minimal upregulation of CD69, suggesting that the upregulation of this molecule in the TCR transgenic T cells was antigen driven and was not due to nonspecific inflammatory stimuli in the liver associated with IV virus administration.

To further elucidate the liver as the site for priming of naïve CD8^+^ T cells, we examined the impact of splenectomy on the response of liver-resident OT-1 T cells to IV virus administration in the adoptive transfer model. To further ensure that CD8^+^ T cells activated within the lymph node compartments were not contributing to the pool of proliferating transgenic T cells in the liver of splenectomized mice, this analysis was carried out in splenectomized and sham splenectomized mice which received monoclonal antibody (Mel-14) to L selectin (CD62L) one day prior to adoptive transfer. This Mel-14 antibody inhibits the migration of circulating naïve T cells into the lymph nodes and prevents T cell activation there. As shown in Fig. 3B, the early proliferative response of naïve CD8^+^ T cells to viral antigen in the liver was unaffected by splenectomy, suggesting that priming of naïve CD8^+^ T cells can occur in the liver.

### CD8^+^ T Cells Activated in the Liver Lead to Defective Differentiation of Effector T Cells

**Figure 4:**
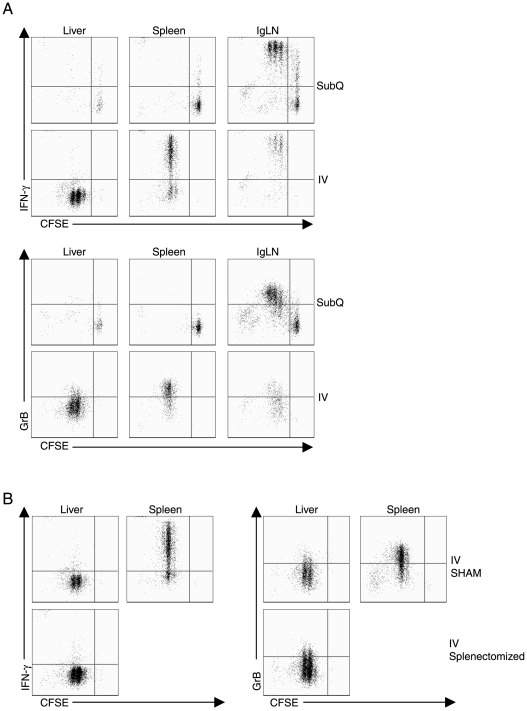
Liver primed CD8^+^ T cells do not differentiate into competent effectors. (A) 2×10^6^ CFSE labeled Thy1.1^+^OT-1^+^CD8^+^ T cells were adoptively transferred into naïve Thy1.2^+^ C57BL/6 mice and then mice were infected with 5×10^8^ PFU Ad-OVA via either SubQ or IV immunization one day later. At 48 hours p.i., liver, spleen, and Ig LN were isolated and leukocytes were stimulated in the presence of OVA peptide and monensin for 5 hours. The ability of dividing Thy1.1^+^CD8^+^ T cells to produce IFN-γ and granzyme-B (GrB) following peptide restimulation was assessed using flow cytometry. The plots are gated on Thy1.1^+^CD8^+^ T cells. (B) 2×10^6^ CFSE labeled Thy1.1^+^OT-1^+^CD8^+^ T cells were adoptively transferred into either splenectomized or sham control Thy1.2^+^ C57BL/6 mice that were treated a day before with Mel-14 Ab. Mice were then infected with 5×10^8^ PFU Ad-OVA via IV administration one day later. At 48 hours p.i., liver and spleen cells were isolated and then restimulated directly ex vivo with OVA peptide for 5 hours in the presence of monensin. The production of IFN-γ and granzyme-B by proliferating Thy1.1^+^CD8^+^ T cells was analyzed by flow cytometry. The plots are gated on Thy1.1^+^CD8^+^ T cells. Data are representative of at least two independent experiments.

The above findings suggested that naïve CD8^+^ T cells activated in the liver are capable of undergoing initial proliferation at this site. However, the expression of at least one marker of T cell activation, i.e. CD25, appeared to be defective in naive T cells activated within the liver environment (Fig. 2, 3). To further explore the impact of T cell induction within the liver environment on CD8^+^ T cell differentiation, we examined the expression of two important CD8^+^ T cell effector molecules, i.e. IFN-γ and granzyme B, in transferred OT-1 T cells in the liver, spleen and inguinal lymph nodes responding to SubQ or IV Ad-OVA virus administration. As Fig. 4A demonstrates, activation/proliferation of naïve T cells following SubQ virus administration was restricted to the inguinal nodes and the proliferating T cells were fully capable of expressing IFN-γ and granzyme B. Following IV virus administration, IFN-γ and granzyme B production were detected in responding T cells stimulated in the spleen and lymph nodes (Fig. 4A). By contrast, naïve T cells activated by virus within the liver failed to upregulate expression of either of these T cell effector molecules (Fig. 4A). When the influence of splenic and lymph nodes derived CD8^+^ T cells are removed from these experiments using splenectomized and Mel-14 Ab treated mice, we similarly observe that liver primed CD8^+^ T cells do not differentiate into potent effectors (Fig. 4B). In companion studies, we evaluated the expression of several other cell surface activation markers. We found that, in contrast to CD8^+^ T cells activated in the spleen or lymph nodes, the majority of naïve CD8^+^ T cells activated within the liver failed to down regulate CD62L (data not shown).

**Figure 5:**
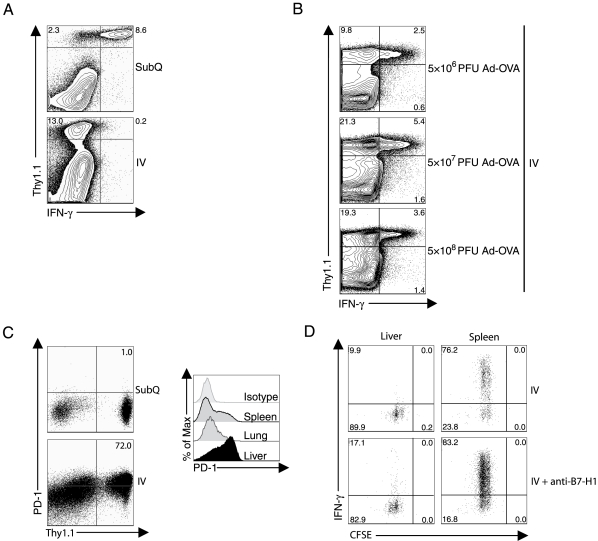
IV adenovirus infection causes a robust defect in CD8^+^ T cell effector function with elevated PD-1 expression in the liver. (A, B) 0.5×10^6^ Thy1.1^+^OT-1^+^CD8^+^ T cells were adoptively transferred into naïve Thy1.2^+^ C57BL/6 mice. These mice were allowed to rest for one day and were then infected with 5×10^8^ PFU Ad-OVA via either SubQ or IV injection (A) or 5×10^8^, 5×10^7^, 5×10^6^ PFU Ad-OVA via IV injection (B). At day 7 p.i., liver leukocytes were isolated and then restimulated directly ex vivo with PMA/ionomycin (A) or OVA peptide in the presence of monensin (B) for 5 hours. The plots are gated on live cells and the numbers represent the percentage of cells within the indicated gates. (C) 2×10^6^ Thy1.1^+^OT-1^+^CD8^+^ T cells were adoptively transferred into naïve Thy1.2^+^ C57BL/6 mice. These mice were allowed to rest for one day and then they were infected with 5×10^8^ PFU Ad-OVA via either SubQ or IV injection. At day 7 p.i., liver leukocytes were isolated and the percentage of Thy1.1^+^CD8^+^ T cells expressing PD-1 was determined. The expression of PD-1 by CD8^+^ T cells in the spleen, lung, and liver following 5×10^8^ PFU Ad-OVA IV infection was also evaluated (right histogram). Both of the plots are gated on CD8^+^ T cells. Data are representative of two independent experiments (*n* = 2/group). (D) Effect of PD-1 blockade on the liver primed CD8^+^ T cell effector activity. 2×10^6^ CFSE labeled Thy1.1^+^OT-1^+^CD8^+^ T cells were adoptively transferred into Thy1.2^+^C57BL/6 mice treated with anti-B7-H1 or control antibody (200 ug per mouse) one day prior to adoptive transfer. The recipient mice were infected with 5×10^8^ PFU Ad-OVA via IV administration one day later. At 48 hours p.i., liver and spleen cells were isolated and then restimulated directly *ex vivo* with OVA peptide for 5 hours in the presence of monensin. The production of IFN-γ by proliferating Thy1.1^+^CD8^+^ T cells was analyzed by flow cytometry and the plots are gated on Thy1.1^+^CD8^+^ T cells.

As demonstrated above (Fig. 1A), viral antigen administration by IV route results in a diminished number of antigen-specific tet^+^ CD8^+^ T cells at days 7 and 14 p.i. compared to SubQ virus inoculation where there is a substantial accumulation of antigen-specific tet^+^ CD8^+^ T cells in the liver. While this discrepancy would most easily argue for a defect in the activation/proliferation of responding CD8^+^ T cells following IV virus administration, our observations on the early response of CD8^+^ T cells in the liver (e.g. Fig. 2 and 3) do not suggest such a defect. Rather the data suggest that IV virus administration and liver priming likely result in a defect in CD8^+^ T cell differentiation into effector cells. To further examine the basis for this potential discrepancy, we analyzed the response of Thy1.1^+^ transgenic CD8^+^ T cells transferred into congenic Thy1.2^+^ recipients at day 7 after administration of virus by SubQ or IV route. Consistent with our earlier findings in Fig. 1A, a large fraction of transgenic Thy1.1^+^CD8^+^ T cells capable of responding to antigenic stimulation with IFN-**γ** production accumulated in the liver on day 7 following SubQ virus delivery. More importantly, a corresponding large number of Thy1.1^+^ transgenic T cells accumulated in the liver following IV virus delivery but these T cells, like activated CD8^+^ T cells analyzed at early time points after antigenic stimulation in the liver (Fig. 2, 3), failed to produce IFN-**γ** (Fig. 5A). In addition, the observed CD8^+^ T cell dysfunction was independent of viral doses administered into mice (Fig. 5B). It suggests that the impaired CD8^+^ T cell function as seen in the IV virus delivery is not due to the high dose of viral antigen administered into the liver.

We next examined whether CD8^+^ T cell dysfunction is associated with the upregulation of PD-1 expression. Functionally exhausted CD8^+^ T cell populations and CD8^+^ T cell death in the liver impair viral clearance during persistent infections including HCV and HBV [33]. The PD-1/B7-H1 inhibitory pathway has been demonstrated to play an important role in the regulation of anti-viral immune responses in the liver [34], [35], [36], [37], [38]. To this end, Thy1.1^+^OT-1^+^CD8^+^ T cells were adoptively transferred into naïve Thy1.2^+^ C57BL/6 mice that were then infected with Ad-OVA via either SubQ or IV immunization route. At day 7 post IV infection, the majority of OVA-specific CD8^+^ T cells in the liver express PD-1 while PD-1 is not expressed by OVA-specific CD8^+^ T cells that migrate to the liver following SubQ immunization (Fig. 5C). In addition, antigen-specific and bulk intrahepatic CD8^+^ T cells generated during IV adenovirus infection express elevated levels of active caspase-3, which serves as an indicator of apoptosis while the liver can support viable antigen-specific CD8^+^ T cells when virus is delivered to other peripheral sites (data not shown). However, treatment of anti-B7-H1 Ab prior to adenovirus infection did not restore the IFN-γ production by CD8^+^ T cells in the liver although it slightly increased the number of IFN-γ^+^CD8^+^ T cells in the spleen compared to mice treated with control Ab (Fig. 5D). It suggests that the PD-1 negative costimulatory pathway might not be directly involved in the induction of CD8^+^ T cells with impaired function in the liver.

### Antigen Dependence of CD8^+^ T Cell Dysfunction in the Liver Environment

Our observations to this point strongly suggest that the liver is a major site of naïve CD8^+^ T cell activation when hepatotropic antigen such as rAd is administered by IV route. Naïve CD8^+^ T cell priming in the liver results in the alteration of CD8^+^ T cell differentiation and expression of effector activities. However, activated CD8^+^ T cells, which accumulate in the liver following SubQ virus administration, do not exhibit a dysfunctional phenotype. Furthermore, IV virus delivery does result in the activation of CD8^+^ T cells within secondary lymphoid organs and the activated CD8^+^ T cells, which remain in that site, appear to be functionally normal (Fig. 2A). Yet if these CD8^+^ T cells within secondary lymphoid tissues, which have been activated in response to IV virus administration and then migrate to the liver, they would appear to also acquire a dysfunctional phenotype.

**Figure 6:**
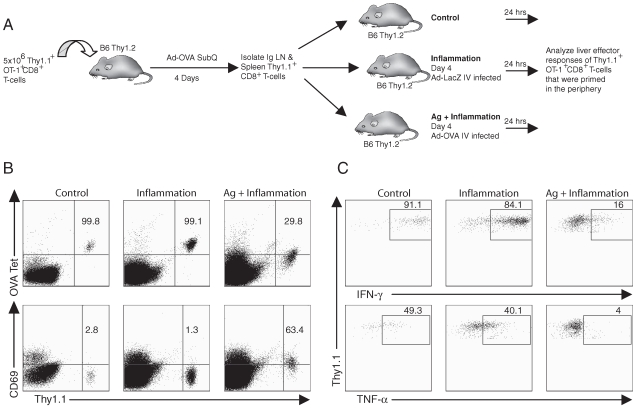
TCR engagement is required for impairment of CD8^+^ T cell effector function in the liver. (A) 5×10^6^ Thy1.1^+^OT-1^+^CD8^+^ T cells were adoptively transferred into Thy1.2^+^ C57BL/6 mice that were then infected with 5×10^8^ PFU Ad-OVA via SubQ injection one day later. On day 4 p.i., spleen and IgLN cells were isolated and Thy1.1^+^ cells were purified. 2×10^6^ Thy1.1^+^OT-1^+^CD8^+^ T cells from day 4 SubQ primed mice were adoptively transferred into Thy1.2^+^ mice that were infected 4 days earlier via IV injection with either 5×10^8^ PFU Ad-LacZ (inflammation group) or 5×10^8^ PFU Ad-OVA (Ag+inflammation group). As a control, 5×10^6^ Thy1.1^+^OT-1^+^CD8^+^ T cells from day 4 SubQ primed mice were adoptively transferred into naïve Thy1.2^+^ mice (control group). (B) At 24 hours post adoptive transfer of SubQ primed effectors, the expression of OVA tetramer and CD69 by intrahepatic Thy1.1^+^OT-1^+^CD8^+^ T cells was analyzed by flow cytometry. The plots are gated on CD8^+^ T cells. The numbers indicate the percentage of adoptively transferred cells positive for OVA tetramer or CD69. (C) Liver cells were also restimulated directly *ex vivo* with OVA peptide for 5 hours in the presence of monensin. The numbers indicate the percentage of Thy1.1^+^OT-1^+^CD8^+^ T cells that produce IFN-γ or TNF-α following peptide restimulation. The plots are gated on Thy1.1^+^CD8^+^ T cells. The data are representative of three independent experiments.

We therefore wanted to determine if, in the liver environment, previously activated functional effector CD8^+^ T cells can be rendered dysfunctional and whether this process was dependent on specific antigen recognition within the liver. To this end, we transferred naïve Thy1.1^+^ OT-1 CD8^+^ T cells into congenic Thy1.2^+^ recipient animals and infected the recipient animals with Ad-OVA virus by SubQ route. Four days after infection, we isolated Thy1.1^+^ T cells from the secondary lymphoid tissues of the recipient mice and then transferred these activated CD8^+^ T cells into Thy1.2^+^ recipient mice, which had been infected with either Ad-OVA or Ad-LacZ by IV route (Fig. 6A). By employing this strategy, we determined whether the effector response of fully activated effector CD8^+^ T cells could be suppressed within the liver environment and whether specific antigen recognition (mediated by IV Ad-OVA administration) or simply acute liver inflammation (orchestrated by IV Ad-LacZ administration) was required to suppress the activated effector CD8^+^ T cells. Activated Thy1.1^+^ OT-1 CD8^+^ T cells adoptively transferred into uninfected recipient mice served as a background control. Upon 24 hours after cell transfer, the Thy1.1^+^ OT-1 CD8^+^ T cells were isolated from the livers of the recipient mice and were analyzed for specific tetramer staining directly *ex vivo* and for the expression of IFN-γ and TNF-α in response to specific antigen stimulation by the *in vitro* intracellular cytokine assay.

The OT-1 T cells isolated from the control (uninfected) livers and from the inflamed livers of mice infected IV with Ad-LacZ virus uniformly bound specific tetramer and expressed IFN-γ and TNF-α in response to peptide stimulation. In contrast, T cells isolated from the livers of mice with the expression of specific antigen by Ad-OVA infection exhibited reduced tetramer staining and defective expression of proinflammatory cytokines compared to the inflammation only and control groups (Fig. 6B, 6C). These results indicate that the liver environment per se or even the inflamed liver environment resulting from virus infection is not sufficient to induce an abortive CD8^+^ T cell response. Rather, the development of this defective response requires specific antigen recognition within the liver.

### Antigen Presentation by Liver Parenchyma Cells Induces Suboptimal Differentiation of CD8^+^ T Cells

Our observations to this point suggest that the liver can serve as a site for the induction of CD8^+^ T cell responses outside the secondary lymphoid tissues. However, naïve T cell activation by antigen within the liver or the encounter of previously activated CD8^+^ T cells with antigen displayed in the liver results in the dysregulation of CD8^+^ T cell effector function. Although the liver contains a variety of CD45^+^ cell types which could serve as APC for naïve or activated antigen specific CD8^+^ T cells, hepatotropic agents like rAd virus may preferentially infect and express antigen in hepatocytes [39], [40], [41]. This consideration prompted us to inquire whether T cell activation in the liver leading to dysregulation of effector function of the activated CD8^+^ T cells may be attributed to the interaction of T cells with antigen displayed on liver parenchymal cells. To explore this possibility, we constructed bone marrow chimeras in which lethally irradiated C57BL/6 mice were reconstituted with allogeneic BALB/c bone marrow. Following reconstitution, these animals contain hematopoietic cells expressing H-2^d^ haplotype MHC class I molecules and liver parenchyma expressing the H-2^b^ haplotype MHC class I molecules. Reconstitution of bone marrow chimera was verified by flow cytometry analysis: the intrahepatic CD11b^+^CD45^+^ cells isolated from Balb/c→B6 chimeras express high levels (>95%) of H2-K^d^, and only a small fraction (∼4%) express H2-K^b^ at levels that are slightly above background. In these mice, only liver parenchyma cells should be able to present OVA_257-264_ epitope to H2-K^b^-restricted OT-1^+^CD8^+^ T cells during Ad-OVA infection. In contrast, the bone marrow derived APCs in the Balb/c→B6 mice chimera mice should express only H2-K^d^ class I molecules and be unable to present cognate OVA antigen to OT-1^+^CD8^+^ T cells during Ad-OVA infection.

**Figure 7:**
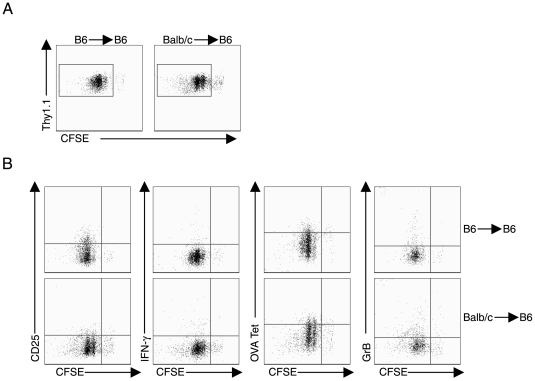
Antigen presentation by liver parenchyma cells induces suboptimal differentiation of CD8^+^ T cells. 2×10^6^ CFSE labeled Thy1.1^+^OT-1^+^CD8^+^ T cells were adoptively transferred into naïve Thy1.2^+^ C57BL/6 mice reconstituted with BalbC or C57BL/6 bone marrow and then the recipient mice were infected with 5×10^8^ PFU Ad-OVA via IV injection one day later. (A) At 48 hours p.i., liver cells were isolated and the proliferative response of OVA-specific CD8^+^ T cells was evaluated. The plots are gated on Thy1.1^+^CD8^+^ T cells. (B) The expression of CD25 and OVA tetramer by proliferating OVA-specific CD8^+^ was evaluated directly *ex vivo*. The ability of dividing Thy1.1^+^CD8^+^ T cells to produce IFN-γ, and granzyme-B following peptide restimulation was assessed using flow cytometry. The plots are gated on Thy1.1^+^CD8^+^ T cells. Data are representative of two independent experiments.

To examine the ability of liver parenchyma cells to present antigen and induce CD8^+^ T cell proliferation, CFSE labeled Thy1.1^+^OT-1^+^CD8^+^ T cells were adoptively transferred into Balb/c→B6 and control B6→B6 mice that were then infected with Ad-OVA by IV immunization route. At 2 days p.i., Thy1.1^+^OT-1^+^CD8^+^ T cells had proliferated to similar levels in both Balb/c→B6 and control B6→B6 mice (Fig. 7A). Thus liver parenchyma cells presenting foreign antigen are able to induce the proliferation of naïve CD8^+^ T cells. Furthermore, CD8^+^ T cells primed by the antigen-presenting liver parenchyma cells fail to differentiate into competent effectors. The dividing T cells do not acquire the ability to produce IFN-γ or GrB following antigen recognition on liver parenchyma cells (Fig. 7B). This suggests that antigen presentation by liver parenchyma cells results in incomplete differentiation of CD8^+^ T cells.

## Discussion

In this report, we examined the impact of foreign (viral) antigen expression and the induction of adaptive immune CD8^+^ T cell response in the liver on the subsequent development of the magnitude and quality of the CD8^+^ effector T cell response. To achieve the liver priming of naïve T cells, we made use of the fact that the hepatotropic adenovirus would preferentially localize and express antigen in the liver when delivered by the IV route. We observed that, in contrast to induction of antiviral CD8^+^ T cell responses and vigorous effector CD8^+^ T cell responses in secondary lymphoid tissues such as lymph nodes and spleen by SubQ or IN virus delivery, induction of CD8^+^ T cell responses in the liver results in a defect in the differentiation of responding CD8^+^ T cells into pro-inflammatory cytokine producing cytolytic effector T cells. In addition, when conventional effector CD8^+^ T cells generated within secondary lymphoid tissues migrated to the target antigen-expressing liver compartment, they also acquire a dysfunctional phenotype resulting in the diminished capacity to express proinflammatory cytokines/cytolytic effector molecules. Finally, studies employing bone marrow chimera mice suggest that the defective effector T cell phenotype exhibited by responding CD8^+^ T cells might be dependent on recognition of foreign antigen displayed on CD45^−^ hepatic parenchyma cells.

The central finding in this report is that intrahepatic stimulation of naïve antigen specific CD8^+^ T cells results in activation and proliferation of the T cells but a failure to express antiviral effector activities, e.g. IFN-γ and granzyme B, characteristic of mature CD8^+^ effector T cells. To some degree, this phenotype parallels the functional “exhaustion” of virus-specific CD8^+^ T cells reported in chronic liver infections with hepatotropic viruses such as HBV and HCV [42], [43], [44], [45]. In those chronic persistent human infections, the apparent exhaustion of effector T cells along with deletion of effector T cells is believed to be linked to the diminished antiviral effector activity and persistence of infection with these agents. Although it has not been examined in this report, the defect in effector activity exhibited by CD8^+^ T cells generated in the liver also appears to adversely affect virus/antigen clearance since antigen (e.g. βgal enzymatic activity) elimination from the site of primary virus inoculation is delayed when the liver is the site of initial infection (J.R. Lukens, unpublished observations). However, the dysfunctional intrahepatic effector T cell response observed in this report is not likely to be specific to recombinant adenovirus because T cells generated to adenovirus infection at other peripheral sites via subcutaneous and intranasal inoculation do not exhibit a similar T cell defect. Furthermore, the recombinant adenovirus with deletion of immunomodulatory proteins, E1, E3, was used in our studies to avoid the interference of host immunity.

Prolonged TCR downregulation has been previously observed as a property of anergic T cells maintained *in vivo* under conditions of high-level antigen persistence [46], [47], [48]. This is likely associated with high dose of virus administration. However, activated CD8^+^ T cells from the livers of mice receiving IV rAd infection also fail to produce IFN-γ or granzyme B in response to the *in vitro* stimulation with PMA/ionomycin (J.R. Lukens, unpublished observations). Importantly, the impairment of CD8^+^ T cell effector activity by IV rAd infection was independent of viral doses administered into mice. In addition, the defect in IFN-γ and granzyme B production and the associated dysfunctional phenotype observed among the CD8^+^ T cells activated in liver did not attribute to the failure of these T cells to recognize antigen because of TCR downregulation. At present, it is yet to be identified the precise mechanism(s) involved in the induction of dysfunctional CD8^+^ T cells primed in the liver.

It is likewise noteworthy that the liver primed activated CD8^+^ T cells have diminished expression of CD25 and increased expression of CD62L. In a survey of other co-stimulatory and inhibitory receptors, we found that the liver primed and localized activated CD8^+^ T cells display elevated levels of the inhibitory receptor PD-1 (Fig. 5C). Although the expression of PD-1 has been linked to the development of the “exhausted” phenotype of CD8^+^ T effector cells and appears to play a role in immune dysregulation mediated by HCV-core protein expressed in the hepatocytes via rAd in this murine model [30], we have been unable to reverse the dysfunctional phenotype of liver primed CD8^+^ T cell effectors by blocking PD-1/PD-1 ligand interaction *in vivo* (Fig. 5D). These findings do not exclude a possible role of PD-1 in the development of the defective effector cell phenotype as recent evidence suggests that blocking of more than one inhibitory receptor on activated T cells may be necessary to restore functionality [49].

The mechanism by which CD8^+^ T cell “priming” in the liver leads to dysfunctional effector cells remains to be determined. As noted above, the liver is recognized as a tolerogenic environment and many factors, e.g. IL-10 expression, have been implicated mechanistically in the establishment and maintenance of this tolerogenic state. Our findings reported here are, to our knowledge, the first to formally demonstrate that the induction of adaptive immune CD8^+^ T cell response within the liver (presumably through presentation of antigen to naïve T cells by liver parenchyma cells) results in the development of a defective effector T cell response. The finding that CD8^+^ T cells activated within secondary lymphoid organs (and therefore undergoing the normal sequence of activation and differentiation into effector cells) are also rendered defective upon specific recognition of antigen displayed by hepatic cells further suggest that the this environment and cognate recognition of antigen by CD8^+^ T cells within the liver profoundly influence the activation/differentiation state of the effector CD8^+^ T cells.

Collectively, our results provide evidence that the liver tissue can support the *de novo* activation of anti-viral naïve CD8^+^ T cells but with incomplete differentiation, and that intrahepatic recognition of antigen by activated CD8^+^ T cells results in dysregulated effector activities. Our study underscores the importance of the liver environment in the generation and control of effector function of the virus-specific CD8^+^ T cells. Interplay between this distinct intrahepatic microenvironment and immune-dysregulatory pathogenic components such as core protein from HCV [50], [51] could lead to the immune evasion by hepatotropic pathogens and promote the establishment of a chronic infection in the liver. Further investigation is warranted to examine in particular the nature of antigen presenting cells and cellular factors that regulate the intrahepatic T cell activation as well as quality of effector CD8^+^ T cell activities at the site of infection. Such information will in turn allow us to manipulate antigen-specific CD8^+^ T cells for a better control of chronic hepatic infections in the human.
